# Editorial: Highlights of IAB IMOSS SEB 2019 Joint Conference

**DOI:** 10.3389/fpls.2021.694765

**Published:** 2021-05-20

**Authors:** Meenu Kapoor, Jeffrey G. Duckett, Stefan A. Rensing, Bernard Goffinet

**Affiliations:** ^1^University School of Biotechnology, Guru Gobind Singh Indraprastha University Sector 16C, Dwarka, India; ^2^Department of Life Sciences, Natural History Museum, London, United Kingdom; ^3^Department of Biology, Plant Cell Biology, University of Marburg, Marburg, Germany; ^4^Ecology and Evolutionary Biology, University of Connecticut, Storrs, CT, United States

**Keywords:** bryophytes, mosses, hornworts, *Funaria*, *Physcomitrium*, *Marchantia*, liverworts

Bryophytes comprise mosses, liverworts, and hornworts that form an ecologically and evolutionarily distinct group from extant vascular plants or tracheophytes and inhabit diverse world-wide geographical locations. Unlike the vascular plants, bryophytes are characterized by a haploid dominant generation (gametophyte) that alternates with a shorter-lived diploid generation (sporophyte). In July 2019 (9th−12th) the International Association of Bryologists (IAB), the International Molecular Moss Science Society (iMOSS), and the Spanish Bryological Association (Sociedad Española de Briología, SEB) jointly held a scientific conference in Madrid, Spain in which bryophyte biologists were invited to share and update the scientific community with their findings related to different topics in bryology that included history, genetics and phylogenetics, taxonomy and systematics, biogeography, checklists, structure and physiology, ecology, climate change, pollution, and conservation. The meeting was attended by 212 researchers of 40 different nationalities. A total of 79 oral lectures were delivered and 85 posters were presented during the 3-day program. Two days prior to the conference (on 7th and 8th July 2019) the IUCN Red-Listing Workshop was conducted by the steering committee of the IUCN SSC Bryophyte Specialist Group that provided training in IUCN methodology to 30 participants of 17 nationalities. The meeting ended with an award ceremony and an excursion. The IAB presented “The Spruce” award and “The Hattori” prize, while iMOSS presented “The Golden Spore” award. “The Spruce” award recognizes an IAB member who has made important contributions to bryology within the first 25 years of his/her/their career and “The Hattori prize” for the best publication in Bryology in the previous 2 years. The winners were Matt Renner (Royal Botanic Garden, Sydney, Australia) and Juan Guerra (University of Murcia, Spain), respectively. The “Golden Spore” award was given to Mitsuyasu Hasebe (National Institute for Basic Biology, Japan) for his pioneering works on evo-devo and the genome of the moss *Physcomitrium patens*. As part of the excursion, on the last day of the meeting (July 12th) participants made a day trip to La Pedriza de Manzanares within the Sierra de Guadarrama National Park near Madrid. A second excursion organized by Modesto Luceño (Pablo Olavide University of Seville) was held after the meeting and extended over 3 days (July 13th, 14th, and 15th) in which 30 participants belonging to 17 different nationalities visited the Sierra de Gredos Natural Park in central Spain.

The oral presentations in the meeting were organized into the broad categories: **Species diversity and evolution, Genetics and functional studies, Development and methods of study**. In each research area selected contributions were presented.

Under the ***Species diversity and evolution section***we present a collection of articles focused on understanding genetic diversity among natural populations and phylogenetic relationships among subspecies. Linde et al. present data on evolution of the *Marchantia polymorpha* complex that clarify the phylogenetic relationships between the three subsp. *montivagans, ruderalis*, and *polymorpha* on the basis of whole genome analysis. This study highlights the significance of hybridization and introgression between subspecies across evolutionary timescales. Biersma et al. present a global phylogeographic reconstruction for the cosmopolitan moss, *Ceratodon purpureus*, revealing a robust biogeographic structure highlighting latitudinal (e.g., pantropical) and bipolar recurrent connectivities. Alonso-García et al. present the first comprehensive phylogeographic and population genetic study of a hornwort, revealing that Southern Appalachian populations of *Nothoceros aenigmaticus*, arose from migration from the neotropical during the Pleistocene and the allopatry of its unisexuality is thus best explained by multiple migrations. Perera-Castro et al. contribute significantly to our understanding of physiological adaptations of Antarctic mosses, by demonstrating that, these mosses have photosynthetic optima at the highest temperatures and achieve positive carbon assimilation by maintaining low respiratory rates at the lowest temperatures. Vendrell-Mir et al. present a non-biased expression analysis of transposable elements (TE) encoded in the *Physcomitrium* (previously *Physcomitrella) patens* genome at different developmental stages and under diverse stress conditions. They suggest that these elements might contribute to variability in genic regions across the genome. Their work also sheds light on TE polymorphism among four different accessions of *P. patens* from Reute (collected close to Freiburg, Germany), Kaskaskia (IL, USA), Villersexel (Haute-Saone, France), and Gransden (Huntingdonshire, UK). The study presented by Spirina et al. explores the homology of filamentous and foliose paraphyllia that develop on the stems of pleurocarpous mosses (i.e., Hypnaceae) based on responses to exogenous application of ABA, proposing a novel hypothesis, whereby not all paraphyllia are alike and that some are in fact homologous to leaves. Sousa et al. explore the effect of alternative models of evolution of chloroplast genes for a broad phylogenetic sampling of streptophytes. They argue, based on inferences from non-synonymous site nucleotide data and amino acid translation data, for the reciprocal monophyly of bryophytes and extant tracheophytes and for Zygnematophyceae that forms the sister group to land plants. Their work provides a robust framework for further understanding of the evolution of early land plants. Maul et al. assess the influence of regional-scale macroclimatic conditions and local factors on the biodiversity of epiphytes and non-epiphytic liverworts in forests in Uganda. They demonstrate that richness of the former ecological group is driven by regional climatic factors, and that of the latter by local microhabitat-specific factors. Based on their study they propose that models relying on macroclimatic factors would only perform well for epiphytic liverworts. Lang et al. present the first population genetic study of a dioicous moss, *Dicranum scoparium*, exhibiting facultative nannandry, that is developing perennial normal sized and ephemeral, epiphytic, dwarf males. They reveal extensive gene flow among female and dwarf male populations, while normal size males tend to be mostly clonal. These authors conclude that each type of males contributes differently but significantly to sexual reproduction, which may explain the persistence of facultative nannandry in this system.

Under ***Genetics and functional studies***, Haas et al. present a genome-wide atlas of Single Nucleotide Polymorphisms (SNP) identified in genic regions of five different accessions of the moss *Physcomitrium patens* and the different laboratory cultivated pedigrees derived over years from the original Gransden accession (collected by H.L.K. Whitehouse in Gransden wood in Huntingdonshire, United Kingdom in 1962). This study shows that prolonged vegetative propagation of plants in laboratories can result in accumulation of deleterious somatic mutations and that annual sexual reproduction may help to purge these mutations. On the basis of identification of characteristic SNPs in different pedigrees and accessions the authors provide a chart with signature SNPs that may facilitate accession/pedigree identification based on RFLP tests. Singh et al. provide insights into the transcriptome landscape of *P. patens* plants harboring mutation in the cytosine DNA/RNA methyltransferase *PpDNMT2* that has been previously shown to be involved in stress recovery. The authors also present quantitative proteomic analysis and shed light on the role of *PpDNMT2* in regulating oxidative stress and maintenance of ion homeostasis by affecting genes and pathways involved in signal perception and transduction.

Under the ***Development and methods section***, Kirbis et al. contrast gene expression in gametophytic and sporophytic generations and across four sporophytic developmental stages of two mosses, *P. patens* and *Funaria hygrometrica*, that exhibit extreme, i.e., reduced and complex, respectively, architecture of their sporophyte. Their analyses suggest that heterochronic expression of major developmental regulators may play a major role in shaping the contrasting complexity of the sporophyte, providing a robust basis for testing whether similar mechanisms drive convergence in sporophyte reduction across mosses. Ignatov et al. reconstruct the pattern of cell divisions in the peristome forming amphithecial layers of the sporangium in a representative of the aperistomate Gigaspermaceae, which are sister to true arthodontous mosses. Their study reveals surprising similarities with the development of the nematodontous peristome of the Polytrichopsida, a lineage that arose from an earlier split in the diversification of mosses.

King et al. describe a semi-automatic and sensitive Object-Based Image Analysis (OBIA) method for long term assessment of vegetation health particularly of plants of short stature such as mosses growing in extreme environmental locations inhospitable for field work. Using this non-destructive methodology, the authors present an assessment of moss health and an evaluation of changes in vegetation cover from 2003 to 2011. Werner et al. present a modified epiGBS protocol for studying DNA methylation changes in genomes of non-model organisms that is cost-effective and that can be useful for laboratories with limited financial resources. Using existing softwares and newly designed algorithms the authors provide proof-of-concept by analyzing methylation in DNA fragments from two almond cultivars *Prunus dulcis* (Mill.) D.A. Webb cv. “Desmayo Largeta” and cv. “Penta” at two different stages during dormancy release.

## Author Contributions

MK wrote the first draft. JD, SR, and BG revised the article. All authors finalized the article.

## Conflict of Interest

The authors declare that the research was conducted in the absence of any commercial or financial relationships that could be construed as a potential conflict of interest.

